# Metagenomic data of microbiota in mangrove soil from Lukut River, Malaysia

**DOI:** 10.1016/j.dib.2024.110155

**Published:** 2024-02-07

**Authors:** Nazariyah Yahaya, Maryam Mohamed Rehan, Nabila Huda Hamdan, Siti Munirah Nasaruddin

**Affiliations:** a Programme of Food Biotechnology, Faculty of Science and Technology, Universiti Sains Islam Malaysia, 71800 Nilai, Negeri Sembilan, Malaysia; b Institut Fatwa dan Halal (IFFAH), Universiti Sains Islam Malaysia, 71800 Nilai, Negeri Sembilan, Malaysia

**Keywords:** Metagenomics whole genome shotgun (mWGS), *Bradyrhizobium*, *Methyloceanibacter*, *Desulfobacteaceae*

## Abstract

The mangrove ecosystem contains sediment microorganisms that play a crucial part in the decomposition of organic matter and the cycling of water and nutrients in the mangrove. Here we present the metagenomics whole genome shotgun (mWGS) sequence data analysis from three soil samples that were collected at the freshwater riverine mangrove at Lukut River, Negeri Sembilan, Malaysia. Data analysis shows different distributions of bacteria of the genera *Bradyrhizobium, Methyloceanibacter* and *Desulfobacteaceae* were detected in soil samples collected at freshwater riverine mangrove. In the data analysis, we report the existence of a large number of Carbohydrate-Active genes in metagenomes collected from mangrove soil. An in-depth exploration of functional annotation analysis based on the KEGG database also showed that the most abundant genes found in these three soils are those that function in carbon fixation pathways, followed by methane, nitrogen, sulfur metabolisms, atrazine and dioxin degradations

Specifications Table

**Table:** 

Subject	Biological Science (Microbiology: Microbiome)
Specific subject area	In this study, we used metagenomic whole-genome shotgun (mWGS) sequencing technology to gain insights into species biodiversity, functions, and pathways within three soil samples collected from the freshwater riverine area of Lukut mangrove. With the rapid advancements in sequencing and informatics technologies, metagenomic investigations using Next Generation Sequencing (NGS) have become increasingly renowned. NGS, as a fundamental strategy for probing community diversity and characteristics, is gaining prominence due to its capacity to generate vast amounts of data and abundant information.
Data format	Raw, Analysed
Type of data	Figures and Charts
Data collection	Soil sediments were collected at a depth of 5 cm from three sampling points within the freshwater riverine mangrove of Lukut River, Negeri Sembilan, Malaysia, during low tide on 20th February 2020. Soil samples were obtained from three locations: soil 1 and soil 2 were collected from a 5 m x 5 m area populated with *Rhizophora mucronata* and *Avicennia officinalis* trees, respectively, while soil 3 was collected from a riverbank near a *Nypa fruticans* tree, approximately 50 m away from soil 1 and soil 2. DNA was extracted from the three soil samples using the QIAGEN Power Soil Pro-Kit (Cat#QIAG-47014). The extracted DNA was then utilized for library preparation and subsequent DNA sequencing using Metagenomic Whole Genome Shotgun (mWGS) technology. The workflow for generating the primary data started with DNA extraction from the mangrove soils. Subsequently, library construction was performed by adding adapters. The library's concentration was determined using Agilent 2100/qPCR, and the procedure concluded with Illumina sequencing. The raw data obtained went through a series of bioinformatics analyses that encompassed data quality control, metagenome assembly, gene prediction, taxonomy annotation and function annotation.
Data source location	Samples collection was conducted within freshwater riverine mangrove of Lukut River, situated in Negeri Sembilan, Malaysia at coordinate 2.5903430, 101.8027310. Lukut River is located in the district of north Port Dickson, Negeri Sembilan, Malaysia and serve as a port for importing and exporting goods such as oil, charcoal and tin via ships. Upstream of Lukut River are the areas of Seremban and Sendayan, characterized by human population density [1]. Lukut itself is a small town with economic activities primarily revolving around small-scale fishing, aquafarming of fish, prawns, and crabs, as well as crop irrigation. The significant of Lukut River lies in its abundant mangrove forests, which serve as a natural habitat for a diversity range of mangrove tree species and gastropod communities [2].
Data accessibility	https://data.mendeley.com/datasets/tzbffkpr9n/2

## 1 Value of the Data

• The data provide valuable insights into the microbial diversity present in the unique mangrove ecosystem of Lukut River. Understanding the composition of microbiota in such environments contributes to our broader knowledge of microbial communities and their ecological roles.
• The metagenomic data allow researchers to study the functional potential of the microbial communities within the mangrove soil. This information can provide insight into the important roles of these microorganisms play in nutrient cycling, organic matter decomposition, and overall ecosystem functioning.
• The dataset opens opportunities for comparative analyses with other mangrove ecosystems globally. By examining similarities and differences in microbiota across various mangrove regions, researchers can identify patterns, drivers, and adaptations unique to each ecosystem.
• The metagenomic data can serve as a resource for discovering novel microbial genes and metabolic pathways with potential applications in biotechnology and environmental management. Identification of genes related to bioremediation, carbon sequestration, and nutrient cycling could have practical implications.
• The metagenomic data can be used as a reference for validating findings in future studies related to mangrove ecosystems, microbial ecology, and environmental genomics.

## 2 Data Description

### 2.1 Metagenomics sequencing

Metagenome sequences were derived from soil samples collected within the freshwater riverine mangrove of Lukut River, Negeri Sembilan, Malaysia. The utilization of the SOAPdenovo protocol for Gut or MEGAHIT for Soil and Water resulted in the assembly of the 34 Mbp genome. This assembly yielded an average of 699,809 scaffolds, with a combined length of 2241 Mbp, and the longest scaffold measuring 0.7 Mbp. The N50 size was calculated at 740 bp.

**Fig. 1:**
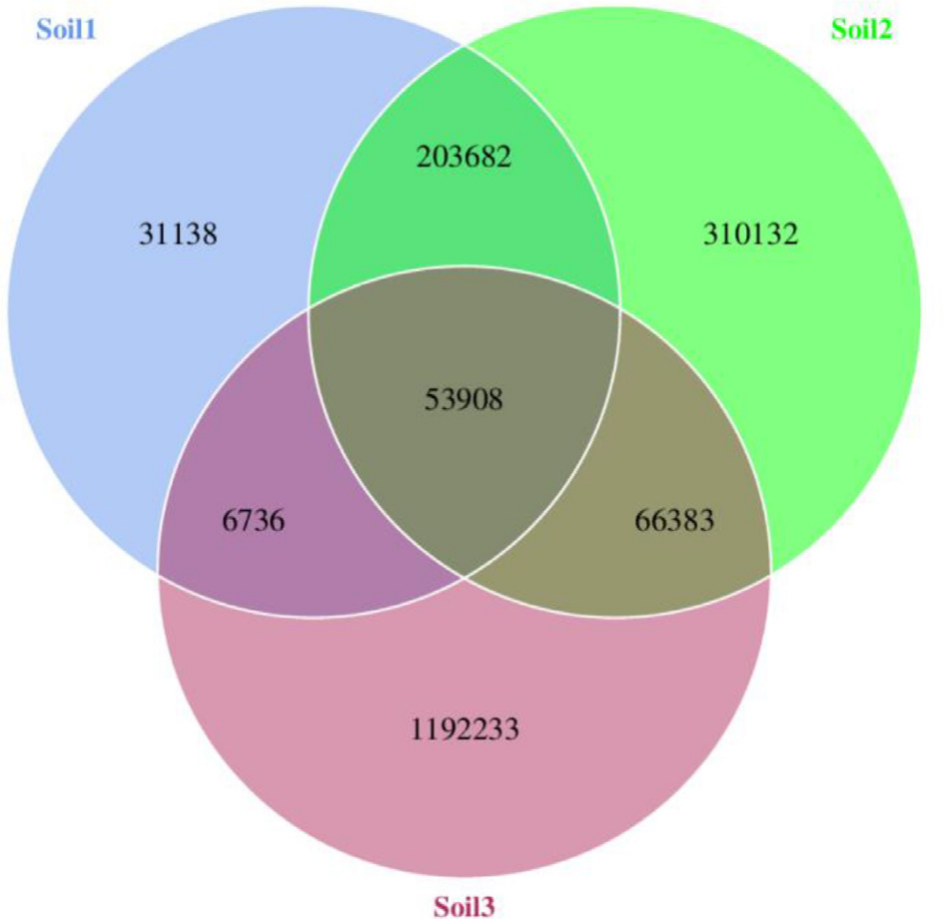
Venn figure shows the number of the common and peculiar genes in soil 1, soil 2 and soil 3.

In this context, the term “genes” refers to non-redundant continuous gene-encoding nucleic acid sequences. The shared and distinct genes among specific samples (soil 1, soil 2, and soil 3) are visually depicted in the Venn diagram (Fig. 1). The effective scaffolds contained a total of 6736 shared genes between soil 1 and soil 3, 203,682 shared genes between soil 1 and soil 2, and 66,383 shared genes between soil 2 and soil 3, which were subsequently utilized for gene prediction (Fig. 1). The overlapping segments denote the common genes shared between samples, while the remaining segments represent the unique genes specific to each sample.

### 2.2 Comparative analysis of functional annotation of bacterial diversity

**Fig. 2:**
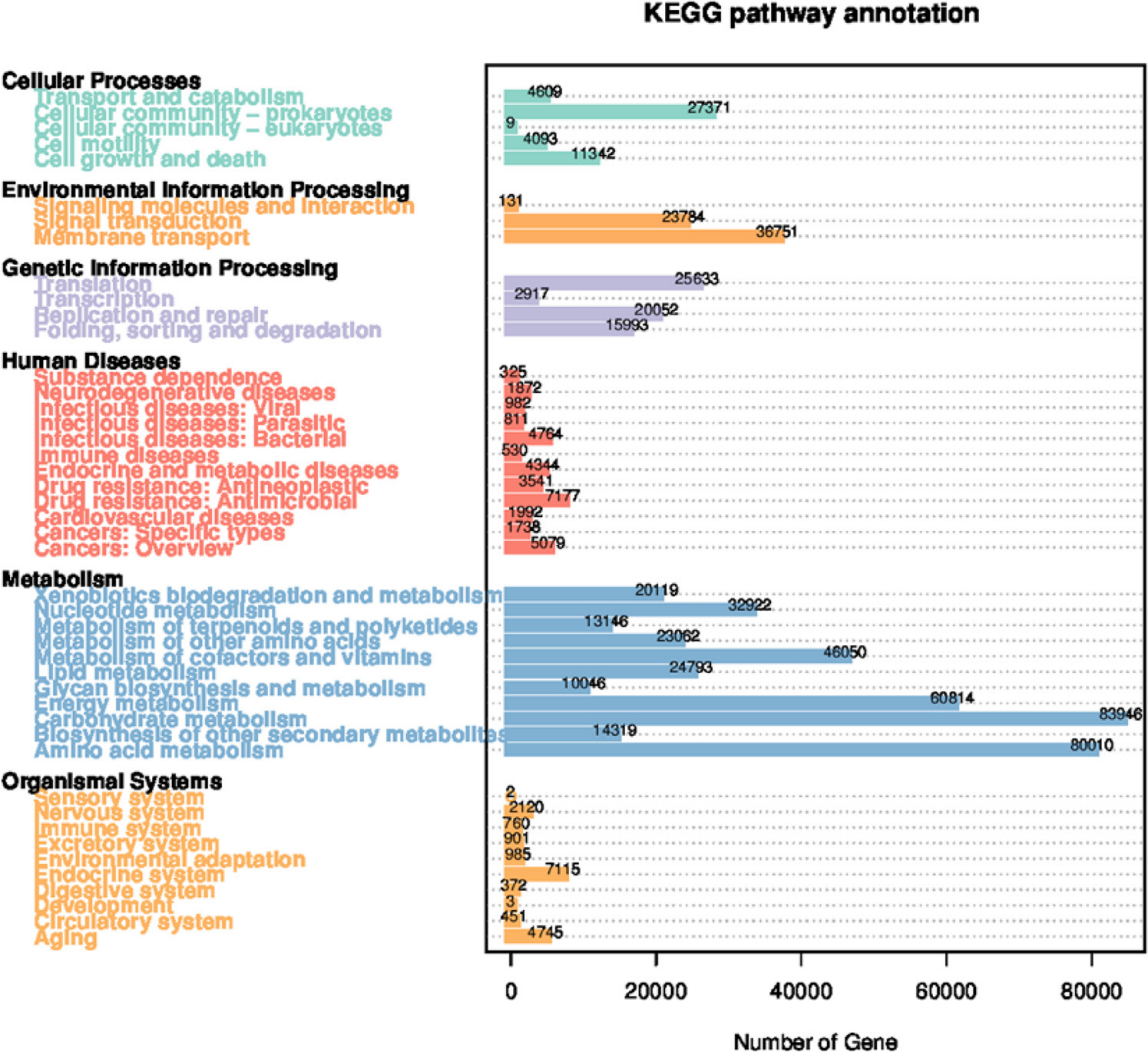
Cartogram of annotated gene number from the unique genes annotation result. The function annotation was performed based on the KEGG database.

Metagenomes offer profound insights into the community's physiology by elucidating the collective functions encoded within the genomes of its constituent organisms. The results of functional abundance, derived from unique gene annotations, are depicted in Fig. 2. According to the KEGG analysis presented in Fig. 2, the core genes prevalent in mangrove soils primarily participate in metabolic pathways. Notably, carbohydrate metabolism claims the highest gene representation, closely followed by amino acid metabolism. Conversely, a smaller gene proportion is attributed to KEGG-annotated human diseases (Fig. 2).

**Fig. 3:**
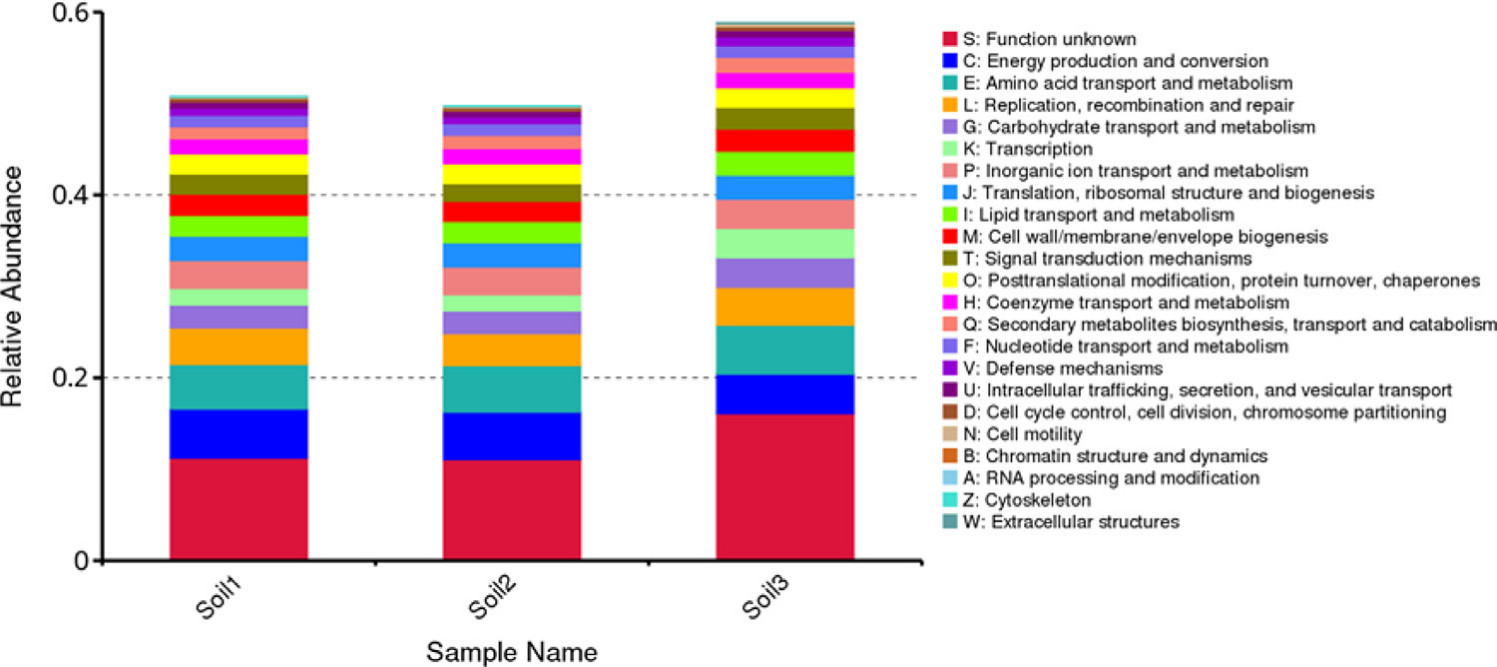
Cartogram of the relative abundance genes found in genome DNA isolated from soil 1, soil 2 and soil 3. The functional annotation was performed based on the eggNOG database.

Furthermore, functional annotation grounded in eggNOG uncovers that genomes isolated from the rhizosphere of mangrove trees' soils largely encompass genes associated with energy production, amino acid transport, metabolism, replication, recombination, repair, as well as carbohydrate transport and metabolism pathways (Fig. 3). However, the principal genes in eggNOG-based functional annotation are categorized under "unknown function" due to a lack of prior research data (Fig. 3).

**Fig. 4:**
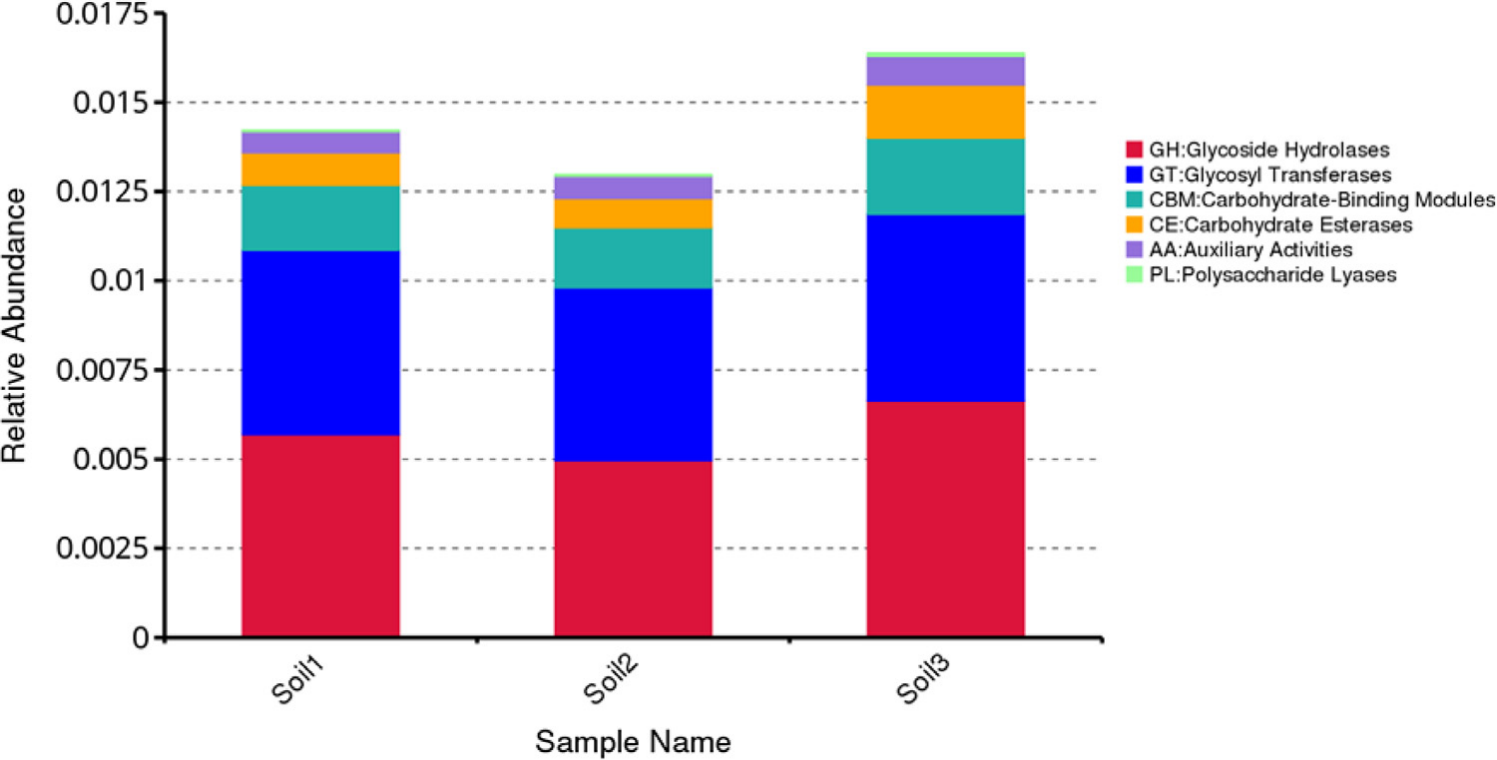
Cartogram of the relative abundance genes found in genome DNA isolated soil 1, soil 2 and soil 3. The functional annotation was performed based on the CAZy database.

The functional annotation based on the CAZy database uncovers six primary CAZy functions: Glycoside Hydrolase (GH), Glycosyl Transferase (GT), Polysaccharide Lyase (PL), Carbohydrate Esterases (CE), Auxiliary Activities (AA), and Carbohydrate-Binding Modules (CBM) present within the collected mangrove soils (Fig. 4). Among these, GH registers the highest gene count, succeeded by GT. The presence of a substantial number of CAZy enzymes and genes related to the carbon fixation pathway in mangrove soil suggests their involvement in the degradation of organic matter, including plant components [3], [4], [5]. The competence and specialization of these bacteria in carbon fixation potentially hold significance in the advancement of bio-composting using plant waste materials.

### 2.3 Functional annotation reveals bacteria signatures and functions

**Fig. 5:**
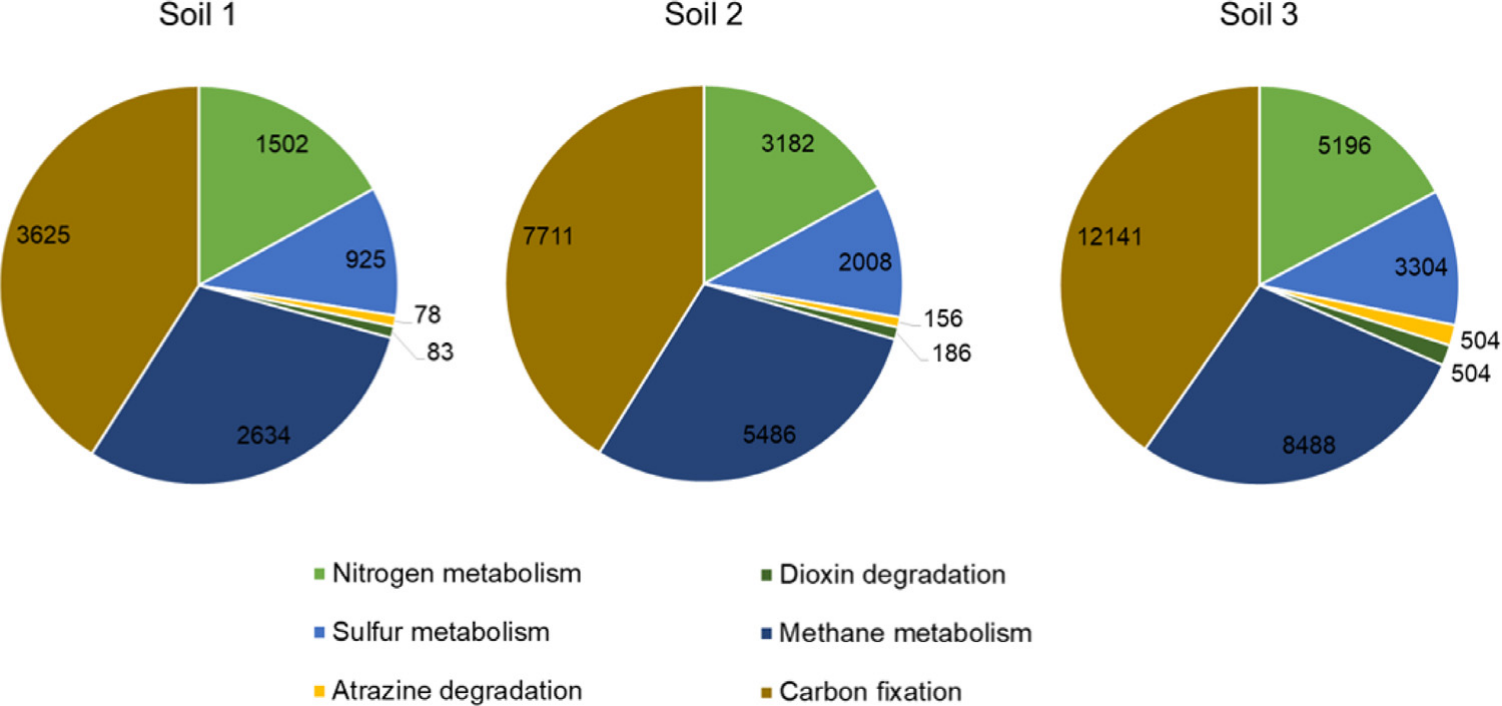
Functional annotation of genes according to KEGG database that involve in nitrogen metabolism, sulfur metabolism, atrazine degradation, dioxin degradation, methane metabolism and carbon fixation pathways found in genome of bacterial species isolated from soil 1, soil 2 and soil 3 of rhizosphere of three mangrove trees species.

A comprehensive exploration of functional annotation analysis based on the KEGG database unveiled the distinctive microbial community within the mangrove soils, characterized by an abundance of genes participating in diverse metabolic pathways. Particularly, carbon fixation emerged as the predominant pathway, followed by methane, nitrogen, sulfur, atrazine, and dioxin degradation pathways (Fig. 5).

**Fig. 6:**
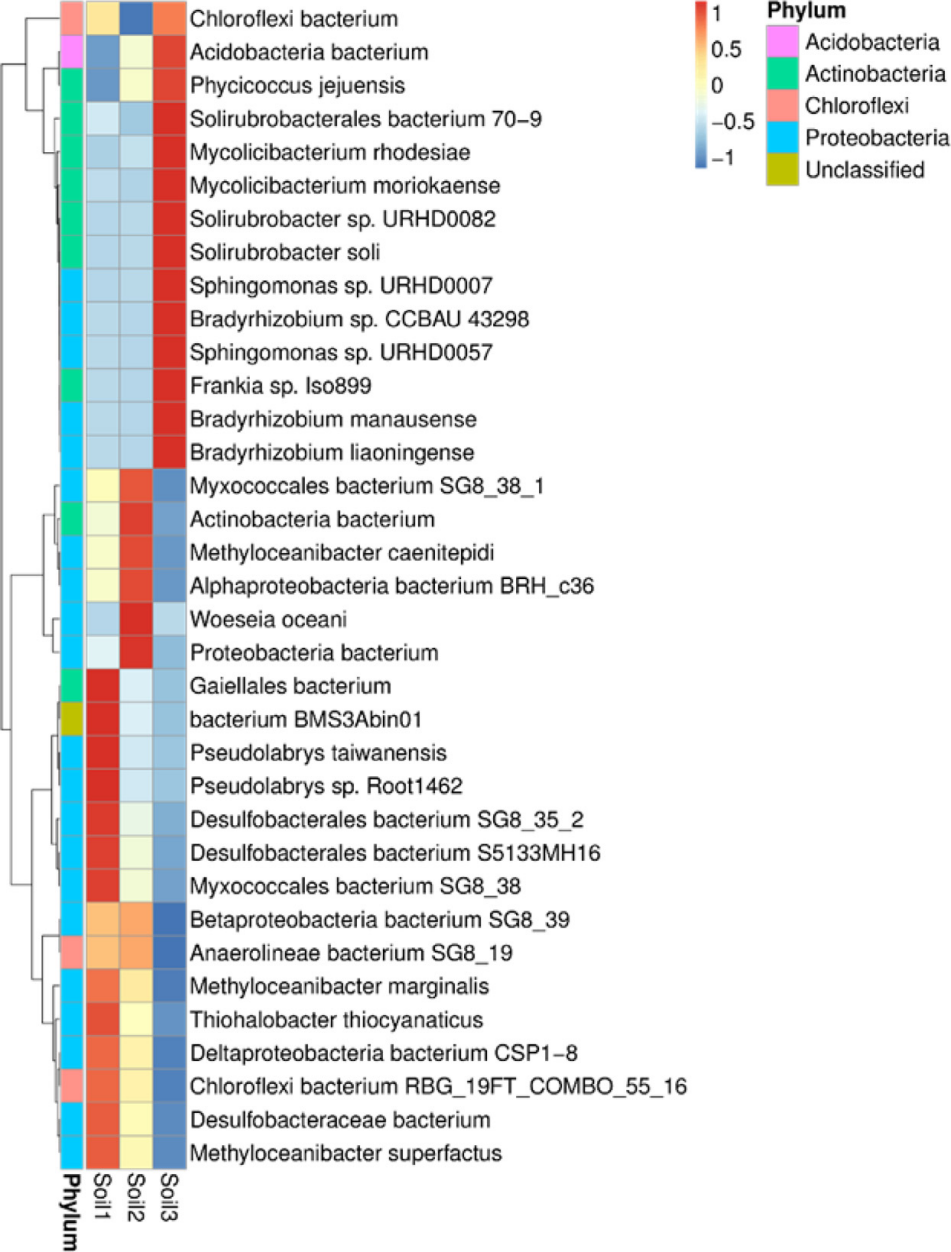
Heatmap analysis of taxonomic diversity of bacteria collected in soil 1, soil 2 and soil 3.

Further analysis pinpointed *Chloroflexi, Gaiellales bacterium*, and *Acidobacteria* as the primary microbial taxa associated with the carbon fixation pathway. The presence of methanotrophic bacteria, including *Methyloceanibacter caenitepidi, M. superfactus*, and *M. marginalis*, was also ascertained (Fig. 6). Furthermore, a multitude of nitrogen-fixing bacteria were identified, encompassing *Chloroflexi bacterium, Acidobacteria bacterium, Actinobacteria bacterium, Frankia* sp.*, proteobacteria bacterium, Betaproteobacteria bacterium, Anaerolineae bacterium, Bradyrhizobium liaoningense, Bradyrhizobium* sp.*, Methyloceanibacter caenitepidi, Methyloceanibacter marginalis, Methyloceanibacter superfactus, Pseudolabrys taiwanensis, Bradyrhizobium manausense, Solirubrobacter* sp.*, Solirubrobacter soli,* and *Phycicoccus jejuensis* (Fig. 6).

Additionally, our investigation revealed the presence of sulfate-reducing bacteria (SRB), including *Thiohalobacter thiocyanaticus, Acidobacteria bacterium, Woeseia oceani, Desulfobacteraceae bacterium, Desulfobacterales bacterium, Mycolicibacterium rhodesiae, Gaiellales bacterium, Deltaproteobacteria bacterium*, and *Myxococcales bacterium*, within the collected soil samples (Fig. 6). The heatmap displayed in Fig. 6 further elucidates the existence of diverse bacterial species classified within the *Actinobacteria phylum, encompassing Phycicoccus jejuensis, Solirubrobacterales bacterium, Mycolicibacterium rhodesiae, M. mariokaense, Solirubrobacter* sp.*, and S. soli* within the collected soils. Furthermore, we detected dioxin degradation bacteria, such as *Chloroflexi bacterium* and bacteria classified under the Betaproteobacteria class, within the mangrove soils.

The roles played by methanotrophic bacteria, sulfate-reducing bacteria (SRB), as well as atrazine and dioxin degrading soil bacteria, are significant in the remediation of contaminated mangrove soil and water. The abundance of methanotrophic bacteria is correlated with increased methane (CH4) concentrations in the soil [6]. SRB's presence is pivotal for the mineralization of sulfur compounds, essential for sustaining mangrove metabolism. The identification of genes associated with the atrazine degradation pathway underscores the contamination of the mangrove ecosystem by chlorine-based herbicides, likely originating from neighboring agricultural activities.

## 3 Experimental Design, Materials and Methods

### 3.1 Sample collection

**Fig. 7:**
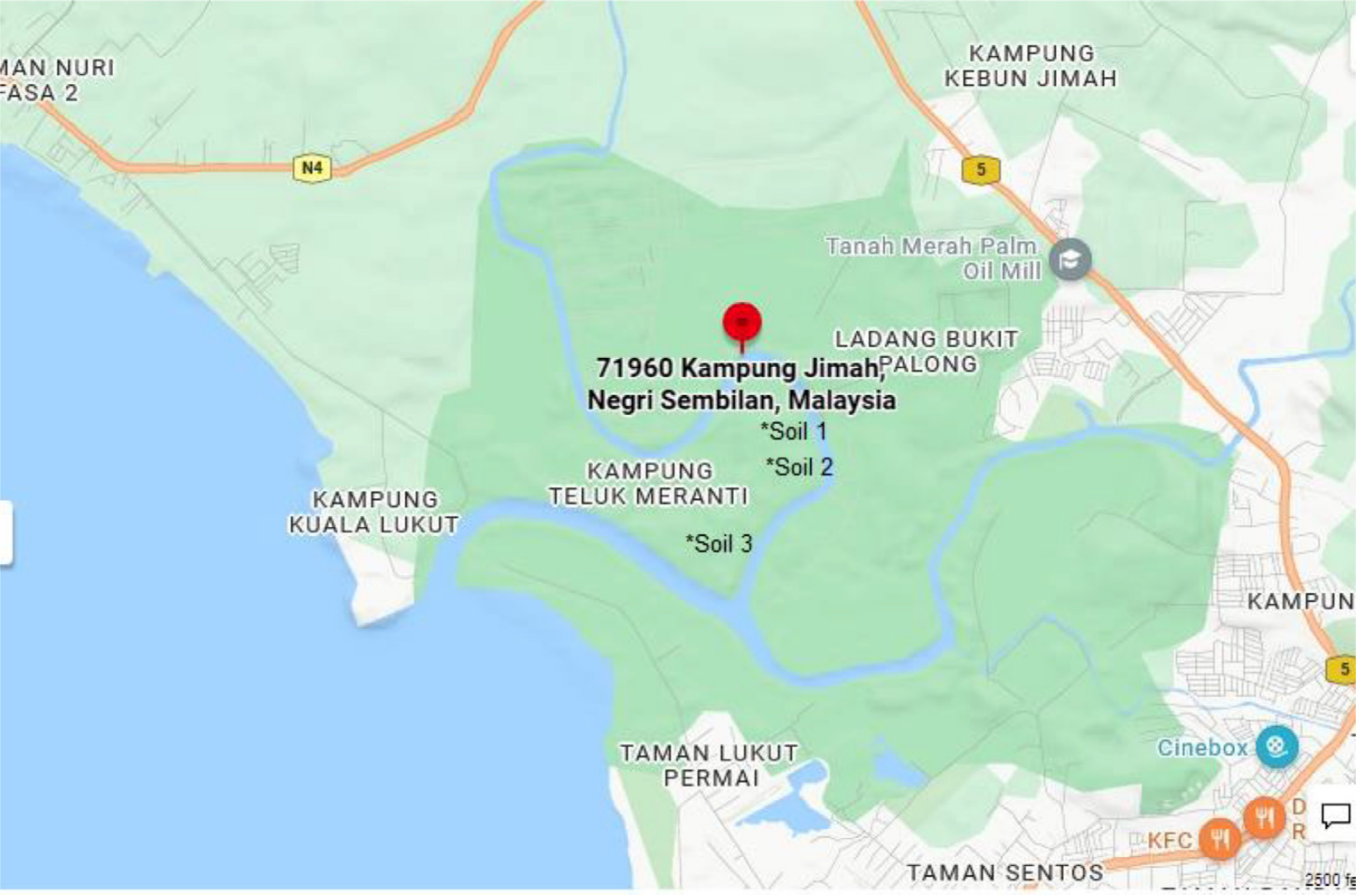
Sampling point at coordinate 2.5903430, 101.8027310, located at Lukut River, Malaysia.

Soil sediments were collected at a consistent depth of 5 cm from three specific locations within the freshwater riverine mangrove of Lukut River, Negeri Sembilan, Malaysia (coordinates: 2.5903430, 101.8027310), as showed in Fig. 7. This collection took place during the period of low tide on the 20th of February 2020. The process involved sampling soil from designated area of a 5 m x 5 m, abundant with *R. mucronata* and *A. officinalis* trees for soil 1 and soil 2. Soil 3 was obtained from a riverbank near the *N. fruticans* tree, approximately 50 m away from both soil 1 and soil 2.

### 3.2 Isolation of DNA from soil samples for metagenomic whole genome shotgun (mWGS).

DNA extraction from the three soil samples was performed using the QIAGEN Power Soil Pro-Kit (Cat#QIAG-47014). The obtained DNA was combined from the three soil samples collected at the same location. DNA purity and integrity were assessed through agarose gel electrophoresis. The Qubit 2.0 fluorometer was used to determine the exact DNA quantity. The resulting DNA was utilized for library preparation and subsequent DNA sequencing, an essential step in the Metagenomic Whole Genome Shotgun (mWGS) process.

### 3.3 Genome sequencing and assembly

To achieve a precise and reproducible distribution of DNA fragments, the Covaris Sonicator was used for mechanical shearing. The metagenome assembly relied on data from Illumina sequencing. Construction and quantification of the metagenomic libraries involved the use of adapters and Agilent 2100/qPCR. Only samples passing quality control were considered for subsequent bioinformatics analyses. In the initial assembly, we adopted the optimized MEGAHIT protocol, known for its effectiveness in reconstructing genomes within microbial communities. This protocol includes outlined steps and parameters to enhance the outcomes when using the MEGAHIT software for preliminary metagenome assembly.

During data quality control, it was observed that the raw data contained percentage of low-quality information. To uphold precision, an intensive regimen of quality control and host filtering was executed, yielding “Clean Data.” Quality control parameters included Clean_Q20 and Clean_Q30, quantifying the percentage of bases with quality score surpassing 20 or 30, respectively. The effective parameter signified the ratio of Clean Data to Raw Data. Scaffolds underwent truncation at “N” to acquire fragments devoid of “N,” referred to as Scaftigs [7,8], representing uninterrupted sequences within scaffolds. To mitigate eukaryotic DNA influence, clean data from all samples were mapped onto assembled Scaftigs using Soap 2.21. Underutilized Paired-end (PE) reads were compiled using specific mapping parameters -u, −2, -m 200.

### 3.4 Gene prediction and abundance analysis

In gene prediction and abundance analysis, scaftigs (with lengths ≥500 bp) went through the process of Open Reading Frame (ORF) prediction using MetaGeneMark [7], encompassing single and mixed samples. To refine the ORF set, trimming was applied to those below 100 nt [7,9]. The dereplication of ORFs and the creation of gene catalogues, the CD-HIT tool [10,11] was employed with parameters -c 0.95, -G 0, -aS 0.9, -g 1, and -d 0. Dereplication involved an identity criterion of 95% and a coverage threshold of 90%, with the longest gene serving as the representative “unigene.” Subsequently, clean data were mapped onto the curated gene catalogue using SoapAligner, with specific parameters set at -m 200, -x 400, and identity ≥ 95%, aiming to assess data quality through alignment efficacy. Finally, gene abundance was computed by considering both the total number of mapped reads and the length of the respective gene. This computation followed a formula in Eq. (1) [12], [13], [14], [15], [16], [17] within a computational framework, ensuring precision and validity.

(1) $$\begin{array}{c} Gk=\frac{rk}{Lk\;}\cdot \frac{1}{\sum_{i=1}^{n}\frac{ri}{Li}} \end{array}$$

### 3.5 Taxonomy and function annotation

To determine the taxonomic affiliations within metagenomic reads, a comparative analysis was conducted by aligning them with a gene family database with taxonomic relevance. This step resulted in annotating each metagenomic homolog with its corresponding taxonomic classification. To gain further insight into the functional aspect of coding sequences, an inference process was employed based on their similarity to sequences in databases such as the Kyoto Encyclopedia of Genes and Genomes (KEGG), the Non-supervised Orthologous Groups (eggNOG), and the Carbohydrate-Active enzymes Database (CAZy). The clustering analysis, visually presented in a heatmap figure, was performed. This analysis was supported by the taxonomic abundance table and function abundance table, providing a comprehensive depiction of patterns and relationships.

## Limitations

Soil 1 and soil 2 were collected from a defined area measuring 5 m x 5 m with *R. mucronata* and *A. officinalis* trees. Soil 3 was obtained from a riverbank near *N. fruticans* tree, approximately 50 m away from soil 1 and soil 2. The DNA was acquired from these three soil samples from the same sampling location.

It is important to note that certain analyses require a minimum number of samples. When the samples count is less than three, conducting PCA, NMDS, CCA / RDA, clustering analysis, and abundance heatmap analysis becomes impractical.

Additionally, it is essential to recognize that statistical analyses, such as Anosim, Metastat, and LEfSe, depend on having at least three biological replicates within a group. Failure to this threshold render the statistical interpretations meaningless, preventing the execution of the analysis itself.

## Ethics Statement

This current work does not involve human subjects, animal experiments, or any data collected from social media platforms.

## Data Availability

Metagenomic data of Microbiota in Mangrove Soil from Lukut River, Malaysia (Original data) (Mendeley Data)

## References

[1] Zainuddin A.H., Aris A.Z., Zaki M.R.M., Yusoff F.M., Wee S.Y. (2022). Occurrence, potential sources and ecological risk estimation of microplastic towards coastal and estuarine zones in Malaysia. Mar. Pollut. Bull..

[2] Nur Anis Fadilah S., Singh H., Hilwani I.Nur (2015). Proc. ISER 10th Int. Conf. Kuala Lumpur, Malaysia, 8th Novemb. 2015.

[3] Dou R., Sun J., Lu J., Deng F., Yang C., Lu G., Dang Z. (2021). Bacterial communities and functional genes stimulated during phenanthrene degradation in soil by bio-microcapsules. Ecotoxicol. Environ. Saf..

[4] Priya G., Lau N.-S., Furusawa G., Dinesh B., Foong S.Y., Amirul A.-A.A. (2018). Metagenomic insights into the phylogenetic and functional profiles of soil microbiome from a managed mangrove in Malaysia. Agric. Gene..

[5] Sun H., Jiang J., Cui L., Feng W., Wang Y., Zhang J. (2019). Soil organic carbon stabilization mechanisms in a subtropical mangrove and salt marsh ecosystems. Sci. Total Environ..

[6] Rzehak T., Praeg N. (2022). Illmer, A standardized and miniaturized method to investigate rhizosphere microorganisms, with a focus on methanogenic archaea and methanotrophic bacteria. Pedobiologia.

[7] Mende D.R., Waller A.S., Sunagawa S., Järvelin A.I., Chan M.M., Arumugam M., Raes J., Bork P. (2012). Assessment of metagenomic assembly using simulated next generation sequencing data. PLoS ONE.

[8] Nielsen H.B., Almeida M., Juncker A.S., Rasmussen S., Li J., Sunagawa S., Plichta D.R., Gautier L., Pedersen A.G., Le Chatelier E., Pelletier E., Bonde I., Nielsen T., Manichanh C., Arumugam M., Batto J.-M., Quintanilha Dos Santos M.B., Blom N., Borruel N., Burgdorf K.S., Boumezbeur F., Casellas F., Doré J., Dworzynski P., Guarner F., Hansen T., Hildebrand F., Kaas R.S., Kennedy S., Kristiansen K., Kultima J.R., Léonard P., Levenez F., Lund O., Moumen B., Le Paslier D., Pons N., Pedersen O., Prifti E., Qin J., Raes J., Sørensen S., Tap J., Tims S., Ussery D.W., Yamada T., Renault P., Sicheritz-Ponten T., Bork P., Wang J., Brunak S., Ehrlich S.D. (2014). Identification and assembly of genomes and genetic elements in complex metagenomic samples without using reference genomes. Nat. Biotechnol..

[9] Qin N., Yang F., Li A., Prifti E., Chen Y., Shao L., Guo J., Chatelier E.Le, Yao J., Wu L., Zhou J., Ni S., Liu L., Pons N., Batto J.M., Kennedy S.P., Leonard P., Yuan C., Ding W., Chen Y., Hu X., Zheng B., Qian G., Xu W., Ehrlich S.D., Zheng S., Li L. (2014). Alterations of the human gut microbiome in liver cirrhosis. Nature.

[10] Li W., Godzik A. (2006). Cd-hit: a fast program for clustering and comparing large sets of protein or nucleotide sequences. Bioinformatics.

[11] Fu L., Niu B., Zhu Z., Wu S., Li W. (2012). CD-HIT: accelerated for clustering the next-generation sequencing data. Bioinformatics.

[12] Karlsson F.H., Fåk F., Nookaew I., Tremaroli V., Fagerberg B., Petranovic D., Bäckhed F., Nielsen J. (2012). Symptomatic atherosclerosis is associated with an altered gut metagenome. Nat. Commun..

[13] Cotillard A., Kennedy S.P., Kong L.C., Prifti E., Pons N., Chatelier E.Le, Almeida M., Quinquis B., Levenez F., Galleron N., Gougis S., Rizkalla S., Batto J.-M., Renault P., Doré J., Zucker J.-D., Clément K., Ehrlich S.D. (2013). Dietary intervention impact on gut microbial gene richness. Nature.

[14] Chatelier E.Le, Nielsen T., Qin J., Prifti E., Hildebrand F., Falony G., Almeida M., Arumugam M., Batto J.-M., Kennedy S., Leonard P., Li J., Burgdorf K., Grarup N., Jørgensen T., Brandslund I., Nielsen H.B., Juncker A.S., Bertalan M., Levenez F., Pons N., Rasmussen S., Sunagawa S., Tap J., Tims S., Zoetendal E.G., Brunak S., Clément K., Doré J., Kleerebezem M., Kristiansen K., Renault P., Sicheritz-Ponten T., de Vos W.M., Zucker J.-D., Raes J., Hansen T., Bork P., Wang J., Ehrlich S.D., Pedersen O. (2013). Richness of human gut microbiome correlates with metabolic markers. Nature.

[15] Oh J., Byrd A.L., Deming C., Conlan S., Kong H.H., Segre J.A. (2014). Biogeography and individuality shape function in the human skin metagenome. Nature.

[16] Zeller G., Tap J., Voigt A.Y., Sunagawa S., Kultima J.R., Costea P.I., Amiot A., Böhm J., Brunetti F., Habermann N., Hercog R., Koch M., Luciani A., Mende D.R., Schneider M.A., Schrotz-King P., Tournigand C., Tran Van Nhieu J., Yamada T., Zimmermann J., Benes V., Kloor M., Ulrich C.M., von Knebel Doeberitz M., Sobhani I., Bork P. (2014). Potential of fecal microbiota for early-stage detection of colorectal cancer. Mol. Syst. Biol..

[17] Michael F.G.K.F., Laurence G., Sabrina S., Stéphane A., Lucie B., Bruno B., Christophe B.J.R.B., Raffaella C., Alison C., d'Ortenzio Fabrizio D.J.R., Jean-Pierre G., Nicolas G., Lionel G., Oliver H.C.N.J., Jean-Louis J., Hervé L.G., Cyrille L., Shruti M., Eric P., Jean-Baptiste R., Simon R., Sébastien S., Eleonora S., Atsuko S.S.M.T., Pierre T., Thomas V., Flora V., Adriana Z., Céline D., Marc P., Sarah S., Stefanie, null null K.L., Peer A.S.G.B., Emmanuel B., Colomban de V., Gabriel G., Hiroyuki O., Stéphane P., Shinichi S.M.B.S., Eric K., Chris B., Fabrice N., Pascal H., Daniele I. (2015). Environmental characteristics of Agulhas rings affect interocean plankton transport. Science.

